# Medical Items

**Published:** 1893-02

**Authors:** 


### *MEDICAL ITEMS.*

---

THE Cooper Medical College, of San Francisco, has adopted the four years course.

---

ANOTHER Keeley Institute, this time at Long Branch, has collapsed from lack of funds and patients.

---

THE English historian, Freeman, who always opposed vaccination, has just died of small-pox.—*Ex.*

---

DR. VIRGIL O. HARDON, of this city, was married to Miss Bertha Wardell, of Bainbridge, Ga., on the 27th of December.

---

THE statue to Dr. Marion Sims in Central Park, New York, is the only statue of a medical man which has been erected in America.

---

TYPHUS FEVER has been epidemic in New York for some weeks. Up to this writing about seventy-five cases have been reported and isolated.

---

A DOCTOR ordered a first-class, self-registering thermometer. A few days after receiving it he wrote that he had had it on ice three days but couldn't get the mercury down. The dealer *shook it down* and the doctor was satisfied.

---

THE city of Bridgport, Conn., has enacted an ordinance prohibiting irregular and unlicensed physicians from practicing in its limits. Connecticut has no medical practice law, and Bridgport is taking measures for its own protection.—*Medical Record.*

MISS MARY GARRETT, of Baltimore, has made a donation of $300,000 for the further equipment of the Johns Hopkins University Medical School. This fund will be used largely in the organization of the departments of anatomy and pharmacology.

---

ONE year ago Dr. Tarnier, of Paris, grieved at the diminishing birth-rate in his country, offered a gift of one hundred francs to every family at Arc-Sue-Fille, his native village, which would contribute one new infant during the year 1892. We are not able now to report the number of successful candidates as all the precincts are not heard from.

---

OUR esteemed neighbor and contemporary, the Alabama *Medical and Surgical Age*, comes out in December greatly enlarged and improved. Dr. Le Grand has labored diligently on his journal and he has made it one of the best published in the South. We take pleasure in congratulating him on the success of his efforts heretofore and in wishing him the same in the future.

---

DR. PAUL GIBIER, of New York, has been treating epilepsy with hypodermic injections of "nervine," a preparation of the gray matter of the brain. Good results are reported to have followed in several cases. The seizures decreased in frequency, attacks of vertigo also decreased, and the patient's intelligence improved. The method is not without some promise, but it may go the way of its Brown-Sequard analogue.

---

ONE of the best medical journals that come to this desk is the *Medical and Surgical Reporter* of Philadelphia. With the January issue it entered upon the forty-first year of continuous publication. Its columns always contain medical matter of a high order of merit, and its editorial pages are occupied weekly with able, conscientious and independent discussions of live and practical subjects. We take special pleasure in congratulating Dr.

Kynett on his late editorial articles on medical education. It is much to be hoped that the words which are now being expended in the interest of this important subject by earnest writers and speakers will accomplish that whereunto they are sent.

---

DR. J. THRUSH has resigned the Chair of Theory and Practice of Medicine in the Cincinnati College of Medicine and Surgery in consequence of ill health, and the vacancy thus created has been filled by the transfer of Dr. E. W. Mitchell from the Chair of Materia Medica and Therapeutics. Dr. G. A. Fackler, Professor of Materia Medica and Therapeutics at the Women's Medical College of Cincinnati, has accepted the appointment to the vacancy created by the transfer of Dr. Mitchell. The College moved into its elegant new building, on Vine Street near Liberty, January 1st. A change has been made with particular reference to the farther development of the Clinical Department of the school.

---

As a recent notice in this journal has informed our readers, the Eleventh International Congress will meet in Rome, Italy, from September 24 to October 1, 1893. By an official letter dated August 22, 1892, and signed by Prof. Guido Baccelli, President, and Prof. E. Maragliano, Secretary-General, Dr. A. Jacobi, of New York, has been directed to form an American sub-committee. Its membership is not yet complete, but on it are already found besides that of the Chairman, the names of Drs. Wm. Osler of Baltimore, S. C. Busey of Washington, N. S. Davis of Chicago, Charles A. L. Reed of Cincinnati, Wm. Pepper of Philadelphia, F. Peyre Porcher of Charleston, James Stewart of Montreal, and Alexander J. C. Skene of Brooklyn, N. Y. In the interest of facilitating the trip to Italy and reducing the expense, arrangements will be made with the steamship companies. According to a communication from the Central Committee, contained in a letter of the Sectary-General's dated September 24, the North German Lloyd proposes to reduce the fare to Genoa by twenty per cent., and that of the return trip by ten per cent. It is expected that still more favorable terms will be secured.

